# Predictors of Diffusing Capacity in Children With Sickle Cell Disease: A Longitudinal Study

**DOI:** 10.3389/fped.2021.678174

**Published:** 2021-05-31

**Authors:** Pritish Mondal, Vishal Midya, Arshjot Khokhar, Shyama Sathianathan, Erick Forno

**Affiliations:** ^1^Division of Pediatric Pulmonology, Department of Pediatrics, Penn State College of Medicine, Hershey, PA, United States; ^2^Department of Environmental Medicine and Public Health, Icahn School of Medicine at Mount Sinai, New York, NY, United States; ^3^Department of Pediatrics, Penn State College of Medicine, Hershey, PA, United States; ^4^Division of Pulmonary Medicine, Department of Pediatrics, University of Pittsburgh School of Medicine, Pittsburgh, PA, United States

**Keywords:** sickle cell disease, DLCO, prediction model, machine learning, pediatrics, pulmonary function test, sickle cell disease, diffusing capacity

## Abstract

**Background:** Gas exchange abnormalities in Sickle Cell Disease (SCD) may represent cardiopulmonary deterioration. Identifying predictors of these abnormalities in children with SCD (C-SCD) may help us understand disease progression and develop informed management decisions.

**Objectives:** To identify pulmonary function tests (PFT) estimates and biomarkers of disease severity that are associated with and predict abnormal diffusing capacity (DLCO) in C-SCD.

**Methods:** We obtained PFT data from 51 C-SCD (median age:12.4 years, male: female = 29:22) (115 observations) and 22 controls (median age:11.1 years, male: female = 8:14), formulated a rank list of DLCO predictors based on machine learning algorithms (XGBoost) or linear mixed-effect models, and compared estimated DLCO to the measured values. Finally, we evaluated the association between measured or estimated DLCO and clinical outcomes, including SCD crises, pulmonary hypertension, and nocturnal desaturation.

**Results:** Hemoglobin-adjusted DLCO (%) and several PFT indices were diminished in C-SCD compared to controls. Both statistical approaches ranked FVC (%), neutrophils (%), and FEF_25−75_ (%) as the top three predictors of DLCO. XGBoost had superior performance compared to the linear model. Both measured and estimated DLCO demonstrated a significant association with SCD severity: higher DLCO, estimated by XGBoost, was associated with fewer SCD crises [beta = −0.084 (95%CI: −0.13, −0.033)] and lower TRJV [beta = −0.009 (−0.017, −0.001)], but not with nocturnal desaturation (*p* = 0.12).

**Conclusions:** In this cohort of C-CSD, DLCO was associated with PFT estimates representing restrictive lung disease (FVC, TLC), airflow obstruction (FEF_25−75_, FEV1/FVC, R5), and inflammation (neutrophilia). We used these indices to estimate DLCO, and show association with disease outcomes, underscoring the prediction models' clinical relevance.

## Introduction

Sickle cell disease (SCD) is a hemoglobinopathy that leads to a chronic inflammatory state resulting in vasculitis, pulmonary fibrosis, and pulmonary hypertension (1). Children with SCD (C-SCD) often suffer from an impaired gas exchange (2), primarily due to chronic airway inflammation, and the association between DLCO and sputum IL-6 level has been reported (2). If untreated, gas exchange abnormalities in SCD may result in chronic hypoxemia, cardiopulmonary morbidity, and poor disease outcomes (3). Chronic hypoxemia in SCD contributes to the pathophysiology of vaso-occlusive crises (VOC) and acute chest syndrome (ACS) (4), and it may also lead to pulmonary hypertension, which can impact life expectancy in this vulnerable population (5, 6). Quantifying the underlying pathophysiologic changes is not feasible in routine clinical practice, and thus gas exchange impairment can be used as a prognostic indicator of disease severity in SCD (7).

The single-breath technique for estimating carbon monoxide uptake, or DLCO, is a standard gas exchange measurement technique (8). In addition to airway inflammation, hypoventilation and anemia also result in DLCO impairment (9–11). Despite its importance, very few studies have been evaluated DLCO in C-SCD. Biltagi et al. (2) reported DLCO impairment in C-SCD compared to controls, and we previously reported an annual DLCO decline of 1.5% in C-SCD (12). Moreover, DLCO impairment in SCD may differ by race/ethnicity (13). However, to date, no studies have focused on the determinants of DLCO in the SCD population. Addressing that knowledge gap could help gain further insight into the origins of impaired gas exchange and prevent related morbidity.

DLCO and lung volumes have a fast rate of decline in SCD (12, 14). This decline is likely multifactorial and could be inter-related and may have prognostic significance. For instance, the relationship between DLCO and FVC has been used to stratify mortality risk in pulmonary hypertension (15); this underscores the importance of studying the predictors of DLCO in SCD, which can lead to pulmonary parenchymal disease, impaired gas exchange and pulmonary hypertension.

Anemia is considered the key determinant of DLCO. Subjects with low hemoglobin typically have under-estimated DLCO. Therefore, for precise interpretation, DLCO should be adjusted for hemoglobin in C-SCD. Association between airflow obstruction and diffusion impairment has been described in adults (16). While gas-diffusion certainly depends on ventilation, it would be important to consider the association between various PFT estimates and DLCO, specially in diseased lungs like SCD, which could be affected by both obstructive and restrictive airway disease. However, several SCD-related studies considered adjusting DLCO for hemoglobin instead of ventilation (17, 18). Our previous study demonstrated the use of impulse oscillometry (IOS) to measure obstructive airway disease (OAD) in C-SCD (12), although a relationship between airway resistance or reactance with DLCO has never been established in C-SCD. Thus, the association between DLCO and spirometric and IOS measures of OAD would be clinically relevant yet relatively unexplored domain. Unlike OAD, restrictive lung disease can be a late manifestation in C-SCD (19), and measures like total lung capacity (TLC) and vital capacity (VC) could be significant predictors of declining DLCO- which is more evident in older C-SCD (2).

In this study, we aim to better understand the predictors of DLCO and their relative importance. Our primary objective was to identify pulmonary function test (PFT) indices and biomarkers that are associated with and predict DLCO in C-SCD and assess their predictive accuracy. Our secondary objective was to determine if estimated DLCO (eDLCO) is associated with clinical outcomes in C-SCD, which would further emphasize the clinical relevance of DLCO.

## Methods

### Study Population

Through retrospective chart review of 140 C-SCD from 2010 to 2020, we identified 51 C-SCD (6–19 years), who were referred to Penn State comprehensive Pediatric SCD clinic to see Pediatric Pulmonology and subsequently performed comprehensive PFTs (spirometry, IOS, plethysmography, and DLCO), along with pertinent laboratory data. The indications for the referral include SCD related chronic lung disease, asthma, and frequent respiratory exacerbation such as wheeze. We also identified 22 race-matched controls (African-American and Hispanic children) who required DLCO primarily to investigate dyspnea of unknown origin but had no cardio-pulmonary or hematological-oncological conditions.

### Predictors of Adjusted DLCO

Percent-predicted DLCO was adjusted for hemoglobin [DLCO/Hb (%)] and was calculated age using sex-specific predictive equations adjusted for age (20). We selected the following potential predictors of DLCO:

- Spirometry measures such as forced vital capacity (FVC), forced expiratory volume in 1 second (FEV1), FEV1/FVC, and the forced expiratory volume between 25 and 75th of FVC (FEF_25−75_) and plethysmography indices like TLC, VC, residual volume (RV), and RV/TLC. NHANES III equations were used to calculate %predicted values.
- IOS estimates of total airway resistance (R5) and reactance (X5, Fres, and AX) were obtained and expressed as %pred using Berdel/Lechtenbörger equations (AX does not have standard reference values) (21). Subjects did not receive bronchodilator therapy for 12 h before the PFTs.
- Laboratory biomarkers: the degree of anemia and biomarkers of hemolysis (LDH, total bilirubin, reticulocyte count) are known to be correlated with SCD-related complications. Biomarkers of systemic diseases, including renal failure, anemia, low fetal hemoglobin (HbF) levels, and leukocytosis, have been reported as significant predictors of mortality in SCD (5). Thus, we included complete blood count (CBC) with differential, HbF, liver and renal function test results, and lactate dehydrogenase (LDH) levels in the preliminary association analyses (**Table 2**).

### Indicators of Disease Severity and Clinical Outcomes

The number of ACS has been reported to have an association with the risk of early death in C-SCD as early as 10 years of age (5, 22). Clinical severity indicators considered in this study include lifetime number of hospitalizations with ACS and VOC; sleep-related nocturnal desaturation (percentage of total sleep time spent with SpO_2_ <90%) (23). Additionally, tricuspid regurgitation jet velocity (TRJV) of 2.5 m/s of higher, measured by echocardiography, was considered as a surrogate marker of pulmonary hypertension (24).

### Statistical Analyses

We used R (3.6.1) and SPSS (25) for data analysis. DLCO estimates falling outside three times the mean Cook's Distance and two-standard deviation of Studentized *t*-values was excluded as outliers. We compared case and control groups with Mann-Whitney U-tests and used Pearson correlations to estimate the association between potential predictors and DLCO. We added a bootstrap correction to Pearson correlation to adjust for non-normality (26).

### Prediction Models

We used variables with a significant association with DLCO/Hb (%) to build the prediction models using two methods, XGBoost, and linear mixed-effects regression. XGBoost is a machine learning instrument that can be used for any type of regression analysis or ranking of the predictors, as programmed by a user-built prediction model (27), while mixed-model is useful for longitudinal data; both approaches can account for variables with repeated measures within participants. Both models were adjusted for potential confounders or effect modifiers such as age, sex, race, hemoglobin genotype (13, 25). Models were also adjusted for hydroxyurea, which increases HbF (28). Finally, models were also controlled for the diagnosis of asthma (yes/no) since it is a major comorbidity in C-SCD and asthma medications (LABA and ICS), which can elevate PFT estimates (29). We built the XGBoost model based on the five-fold cross-validation (CV) method. Subjects were randomly divided into five equal groups; four of those five groups were selected at a time as training data and the remaining one as test data, and the process was repeated five times. Based on the results, the predictors of DLCO were selected, and the algorithm was built. Predictors were ranked based on their relative importance determined by “gain” measure in XGBoost and by *p*-values in the linear mixed model. To quantify both models' performance in terms of predictive accuracy, we calculated the mean absolute percentage error (MAPE) and the correlation coefficient between measured and eDLCO. MAPE values of <10% and between 10 and 20% were considered as “excellent” and “good” forecasting, respectively (30). Further details on predictors and model selection are described in Supplementary Data 1.

### Association Between DLCO and Clinical Outcome Measures of SCD

To confirm the prognostic importance of DLCO, we analyzed its association with SCD clinical outcomes using linear regression, adjusted for age and sex. For the correlational analyses between lifetime events (numbers) of VOC/ACS and DLCO, we used the median values of DLCO for the subjects with multiple data points. We also conducted correlation analyses between measured DLCO and other disease severity indicators, including TRJV and nocturnal desaturation. Then we used both the prediction models to calculate eDLCO, and further analyzed the association between eDLCO values and outcome measures using linear regression to cross-examine the accuracy and clinical relevance of the prediction models.

### Validation of the Prediction Model

Leave-one-out performance (LOOP) was used to cross-validate XGBoost model (31). Using “LOOP” function, predicted DLCO was estimated for every single observation while the remaining data was used to train the algorithm. This process was repeated 112 times (excluding 3 outliers). The forecast's strength was estimated with MAPE and the Pearson correlation coefficient between observed vs. predicted DLCO.

## Results

Fifty-one C-SCD performed a total of 115 DLCO measurements (range: 1–6/subject). The cohort of C-SCD was comprised of 41 African-American and 10 Hispanic children, with 29 males and 22 females, respectively. HbSS (41/51) was the most common genotype, followed by HbSC (8/51) (Table 1). Mean (SD) DLCO/Hb (%) was 87.9 (17.2)%; there were no differences in DLCO between hemoglobin genotypes (HbSS vs. HbSC; *p* = 0.74) or the two racial/ethnic groups (African American vs. Hispanic; *p* = 0.82). At the time of PFTs, mean (SD) age and height of the study participants were 13.0 (3.7) years and 150.1 (17.6) cm, while 91, 50, and 17% of C-SCD were on hydroxyurea, ICS, and LABA, respectively. The average number of lifetime ACS/VOC episodes was 3.16 (2.63). Results for SCD biomarkers are summarized in Supplementary Table 1.

**Table 1:** Baseline characteristics of the study cohort.

**Variables**	**N**	**Median (25th-75th pct)**	**N**	**Median (25th-75th pct)**	***p*-value**
Age (years)	112	12.4 (9.3–15.5)	22	11.1 (9.4-12.9)	0.052
Male/female	51	29/22	22	8/14	0.107^#^
African American/Hispanic	51	41/10	22	14/8	0.127^#^
Genotype (HbSS/HbSC/other)	51	41/8/2	22	n/a	n/a
DLCO-Hb (%)	112	87.0 (74.4–99.6)	22	94.8 (80.7–109.0)	0.034
DLCO/VA	111	5.48 (5.8–6.2)	22	6.33 (5.6–7.0)	<0.001
FVC (%)	97	91 (71–111)	19	106.0 (89.5–122.5)	0.001
FEV1 (%)	96	88 (77–99)	19	99.0 (86.5–111.5)	0.011
FEV1/FVC	97	83.8 (78.3–89.3)	19	81.0 (78.5–83.5)	0.089
FEF_25−75_ (%)	97	74 (64–84)	19	78.0 (61.5–94.5)	0.749
TLC (%)	111	86 (81.5–90.5)	22	97.5 (86.5–108.5)	0.002
VC (%)	111	88 (79.8–96.3)	22	101. (88.0–114.0)	0.002
RV/TLC	111	26 (21.5–30.5)	22	27 (21–33)	0.648

*Mann-Whitney U tests were used to compare between children with SCD and the control subjects for continuous variables and*

# *chi-quare tests for binary/categorical variables. The controls did not have pre-existing hematological conditions. For those children, DLCO was adjusted for normal hemoglobin if hemoglobin level was unavailable. P-values < 0.05 were considered significant. N, number of subjects; pct, percentile*.

### Control Group

The mean age of the controls was 10.8 (2.9) years. C-SCD had lower DLCO/Hb (%pred), FVC (%pred), FEV1 (%pred), TLC (%pred), and VC (%pred) compared to controls (Table 1). Race/ethnicity and gender distributions were not statistically different between cases and controls (Table 1).

### Evaluation of DLCO Predictors

The correlations between hemoglobin-adjusted DLCO with PFT estimates, anthropometrics, and biomarkers are presented in Table 2. DLCO was moderately and positively correlated with FEV_1_ (%pred), FVC (%pred), FEV_1_/FVC, FEF_25−75_ (%pred), TLC (%pred); and inversely correlated with R5Hz (%pred) and with peripheral blood neutrophilia (either a percent of WBC or as absolute counts). Among the PFT estimates, FEV1, FEV1/FVC, and FEF_25−75_ represent obstructive airway disease, while a decline TLC and FVC often indicate restrictive ling disease. Increased total airway resistance (R5Hz) can lead to decreased airflow exhalation, thus widely used in asthmatic children who also have diminished spirometric indices representing obstructive airway disease. Aspartate Aminotransferase (AST), total bilirubin, and LDH had a positive correlation with hemoglobin-adjusted DLCO. However, those associations were driven by the strong correlation between the laboratory results and hemoglobin (Supplementary Table 2) (32), and thus they were not included in further analyses to prevent over-adjustment bias.

**Table 2:** Correlations between DLCO and clinical characteristics.

**Independent variables**	**Correlation coefficient**	**95% confidence interval**	***p*-value**
**Pulmonary function tests**			
FEV1 % predicted	0.378	(0.207, 0.526)	<0.001
FEF_25−75_ % predicted	0.410	(0.243, 0.553)	<0.001
FVC % predicted	0.305	(0.126, 0.464)	0.001
R5Hz % predicted	−0.246	(−0.413, −0.063)	0.009
FEV1/FVC	0.243	(0.060, 0.410)	0.010
TLC % predicted	0.214	(0.030, 0.384)	0.023
RV/TLC	−0.137	(−0.314, 0.050)	0.150
X5Hz % predicted	0.016	(−0.170, 0.201)	0.869
AX	−0.014	(−0.199, 0.172)	0.886
**Anthropometry**			
Height	−0.142	(−0.319, 0.045)	0.135
Age	−0.115	(−0.294, 0.072)	0.228
Weight	−0.114	(−0.293, 0.073)	0.232
**Biomarkers**			
Neutrophil (% of WBC count)	−0.316	(−0.474, −0.138)	<0.001
AST	0.258	(0.076, 0.423)	0.006
Total bilirubin	0.250	(0.068, 0.417)	0.008
Neutrophil count (ANC)	−0.205	(−0.377, −0.021)	0.030
Lactate dehydrogenase (LDH)	0.198	(0.013, 0.370)	0.036
Hemoglobin F	−0.012	(−0.197, 0.174)	0.898
Absolute reticulocyte count	0.004	(−0.182, 0.189)	0.970
WBC	−0.097	(−0.278, 0.090)	0.307
Platelets	−0.090	(−0.271, 0.098)	0.348
ALT	−0.124	(−0.302, 0.063)	0.194
BUN	−0.109	(−0.289, 0.078)	0.252
Creatinine	−0.069	(−0.251, 0.118)	0.471

*Pearson correlation between Hb-adjusted DLCO (%pred) and pulmonary function test results, anthropometrics, and lab estimates. Correlation analyses recognized the variables with significant association with DLCO. P-values < 0.05 were considered significant*.

### Prediction Models

The ML Tool XGBoost identified FVC (%), neutrophil (%), and FEF_25−75_ (%) as the top three predictors (Table 3). The MAPE for the model was 1.81%, indicating excellent performance. Similarly, the linear mixed-effects regression analysis identified FVC (%), neutrophil (%), and FEF_25−75_ (%) as the top three predictors for adjusted DLCO (Table 2). The regression model reproduced the exact rank list of six predictors as the XGBoost model (Table 3). MAPE between measured and eDLCO for the mixed-model was 9.1%, inferior compared to XGBoost (Supplementary Figure 1).

**Table 3:** The ranking of the predictors of DLCO.

	**XGBoost model**		**Adjusted linear mixed effect regression analysis**				
**Variables**	**Rank**	**Gain**	**Rank**	**Regression coefficient**	**Standard error**	***t*-value**	***p*-value**
FVC % pred	1	0.213	1	0.278	0.102	2.724	0.008
Neutrophil (% of WBC count)	2	0.181	2	−0.274	0.116	−2.363	0.020
FEF_25−75_ % pred	3	0.178	3	0.182	0.087	2.090	0.039
FEV1/FVC	4	0.159	4	0.212	0.316	0.671	0.503
R5Hz % pred	5	0.158	5	−0.037	0.060	−0.621	0.536
TLC % pred	6	0.110	6	0.017	0.108	0.155	0.877

*The ranking of the predictors of hemoglobin-adjusted DLCO, based on the “Gain” measure in XGBoost and on p-values (significant at < 0.05) in the adjusted regression analysis*.

### Measured and Estimated DLCO vs. Outcome Measures

Measured DLCO was significantly associated with the number of lifetime VOC/ACS events and TRJV (Table 4), but not with nocturnal desaturation (*p* = 0.13). After adjusting for age and sex, each 1% decrease in adjusted DLCO was associated with 0.08 more lifetime ACS/VOC events and 0.009 m/s higher TRJV (Table 4). eDLCO, obtained from our predictive models, was also significantly associated with AOC/VOC events and TRJV (Table 4): after adjusting for age and sex, each 1% decrease in eDLCO was associated with 0.08–0.10 more lifetime ACS/VOC events and with 0.009–0.014 m/s higher TRJV (Table 4).

**Table 4:** Adjusted analysis of DLCO vs. SCD clinical outcomes.

**Clinical outcome**	**Adjusted linear regression analyses**		
	**Regression coefficient**	**95% CI**	***p*-value**
**DLCO vs. SCD crises**			
Measured DLCO	−0.075	−0.120 to −0.030	0.002
Estimated DLCO (XGBoost)	−0.084	−0.134 to −0.033	0.002
Estimated DLCO (mixed-model)	−0.102	−0.170 to −0.034	0.004
**DLCO vs. TRVJ**			
Measured DLCO	−0.009	−0.017 to −0.001	0.029
Estimated DLCO (XGBoost)	−0.009	−0.017 to −0.001	0.038
Estimated DLCO (mixed-model)	−0.014	−0.025 to −0.003	0.014
**DLCO vs. nocturnal desaturation**			
Measured DLCO	0.005	−0.002 to 0.012	0.137
Estimated DLCO (XGBoost)	0.006	−0.002 to 0.013	0.121
Estimated DLCO (mixed-model)	0.013	0.001 to 0.125	0.031

*Linear regression analyses demonstrating a significant association between DLCO (measured and estimated) with outcome measures of SCD, except nocturnal desaturation. Regression models were adjusted for age and sex. Hemoglobin-adjusted DLCO (%) was included in the study analyses. SCD crises were defined as the number of lifetime events (vaso-occlusive crisis and acute chest syndrome that required hospitalization). TRJV (m/s) was considered as a marker of pulmonary hypertension. Nocturnal desaturation was defined as the percent of total sleep time spent with SpO_2_ <90%. P-values < 0.05 were considered significant*.

### Validation of the Prediction Model

We tested the strength of the prediction model using the LOOP method. Estimated DLCO (mean ± SD) was 87.9 ± 17.18 compared to a measured DLCO of 87.79 ± 10.87, with good forecasting (MAPE of 17.3%) and significant correlation (*r* = 0.40, *p* < 0.001) between two groups (Supplementary Figure 2).

## Discussion

In this study in children with SCD, we show that PFT estimates representing OAD (FEF_25−75_, FEV1/FVC, R5%), restrictive lung disease (FVC%, TLC%), and biomarkers of inflammation (neutrophil%) were associated with DLCO; and that models built on those six variables can calculate “estimated DLCO (e-DLCO)” with precision. Moreover, we demonstrate that lower DLCO and e-DLCO are significantly associated with worse clinical outcomes, including more frequent ACS/VOC events and evidence of pulmonary hypertension. These results advance our understanding of factors associated with impaired gas exchange in SCD.

Most pediatric SCD centers in the US do not offer a multi-disciplinary clinic, and PFTs (including DLCO) are not always a part of standard of care in C-SCD. Clinical status can change rapidly in these children, and PFTs along with other biomarkers should be obtained especially in children with frequent complications, to monitor the clinical deterioration of the cardiorespiratory system. Considering the prognostic significance of impaired gas-exchange, DLCO should be incorporated into a standard of care in C-SCD with frequent SCD crises, and in-depth clinical research on DLCO is necessary. American Society of Hematology (ASH) guidelines, 2019, recommends obtaining PFTs in SCD patients with various respiratory symptoms even if they are at their steady state (33). ASH acknowledges that the usefulness of routine PFT is unknown because of the lack of research, and thus it does not recommend PFTs for every SCD patient. However, ASH further suggests that if the PFTs are obtained, it should be a comprehensive study including lung volumes and DLCO, in addition to spirometry (33).

We found that C-SCD significantly lower PFTs than their peers without SCD, consistent with previous studies that have reported impaired lung function in SCD (2, 9). On the other hand, we did not find associations between biomarkers of systemic involvement and DLCO, as has been described in adult SCD literature (17). This could be partially explained by differences in disease severity or progression in adults with SCD compared to younger populations.

OAD is a relatively early phenomenon in SCD lung involvement, and it can be measured by spirometry and IOS. We found that FEF_25−75_ (%) and FEV1/FVC were positively correlated with DLCO, indicating an association between OAD and impaired gas diffusion in C- SCD. One of the novel aspects of this study was our ability to examine the association between IOS estimates and DLCO. Although a relationship between IOS estimates and DLCO has never been studied in SCD, a negative correlation between airway resistance and DLCO has been reported in adults with idiopathic pulmonary fibrosis (34). With age, airway resistance increases (12), and DLCO (%) decreases in C-SCD (2); thus, the significant inverse correlation between R5 (%) and DLCO (%) may represent a parallel deterioration in gas diffusion and airway obstruction.

Restrictive airway disease is a relatively late phenomenon in youth with SCD (9). As the disease progresses, lung volumes and DLCO simultaneously decline due to recurrent inflammation and progressive pulmonary fibrosis (17, 35, 36). The alveolar ventilation and the rate of uptake of CO (KCO) determines the gas exchange. However, SCD is path-physiologically unique and complex, since the diseased lungs could be affected by obstructive as well as restrictive changes, and their relative impact on DLCO is unknown. The positive correlation we report between DLCO and lung volume indices such as FVC (%) and TLC (%) further indicate that gas diffusion could also be influenced by restrictive lung disease, and these associations may start in childhood, even with apparently normal lung functions. Recurrent SCD crises lead to parenchymal disease and impaired gas diffusion (2, 37). Neutrophil activation generates extracellular traps, triggers endothelial activation and pro-inflammatory pathways in SCD (38), resulting in thromboembolism in the pulmonary microvasculature, triggering VOC (39). Thus, neutrophilia may indicate disease severity, and it has been recognized as a predictor of mortality in SCD (5). We found that neutrophilia (both count and percent) had an inverse correlation to DLCO, and neutrophil (%) was among the top three predictors of DLCO. Absolute neutrophil counts have been reported to have an inverse correlation with DLCO in the general population (40), but to our knowledge, this is the first report associating neutrophilia with impaired gas exchange in C-SCD.

The study has several limitations that should be acknowledged. It was a retrospective, single-center study, and thus we could not evaluate the effect of center-level practices on our results. Since an external cohort was not available, further studies will be needed to validate our findings. We lacked racial and genotypical diversity in the study population, although this is probably fairly representative of the SCD population as a whole. Most of the subjects were in their early teens and had stable lung function, and therefore we could not extrapolate to younger or older ages; the predictor rank list may have been different in young children or adults with advanced SCD lung disease. We used TRJV as a surrogate marker of pulmonary hypertension. However, TRJV was a screening tool. To diagnose pulmonary hypertension, right heart catheterization was necessary, which was beyond the scope of this analysis and would merit evaluation in future, prospective studies. At the same time, our study has several strengths. While diffusing capacity is an important biomarker of SCD lung pathology and is associated with clinical outcomes, diffusion limitation and its probable predictors have not been well-studied in C-SCD. We had repeated longitudinal comprehensive PFT data for the cohort. We used two different statistical approaches; while one was more accurate than the other in estimating DLCO, both selected the same predictors, which included easy to obtain spirometric and laboratory values. Finally, both measured and estimated DLCO were associated with SCD clinical outcomes and successful cross-validation of the XGBoost model further added reliability to the prediction model (30).

In conclusion, in a cohort of children with SCD, we report several markers associated with impaired gas exchange, including PFT estimates representing restrictive lung disease (FVC), obstructive airway disease (FEF_25−75_), and inflammation (neutrophils). DLCO was associated with SCD severity indicators, and we were able to use simple predictors to calculate eDLCO, which was significantly associated with disease outcomes. This underscores the clinical relevance of our prediction models and could help to identify children at risk.

## Data Availability Statement

The raw data supporting the conclusions of this article will be made available by the authors upon request, without undue reservation.

## Ethics Statement

The studies involving human participants were reviewed and approved by Penn State College of Medicine Institutional Review Board. Written informed consent from the participants' legal guardian/next of kin was not required to participate in this study in accordance with the national legislation and the institutional requirements.

## Author Contributions

PM: PI of the study, contributed in building study protocol, data collection, statistical analyses and interpretation, manuscript writing, and final draft approval. VM: contributed in building study protocol, statistical analyses and interpretation, manuscript writing, and final draft approval. AK and SS: contributed in data collection, statistical analyses and interpretation, manuscript writing, and final draft approval. EF: contributed in building study protocol, statistical analyses, data interpretation, manuscript writing, and final draft approval. All authors contributed to the article and approved the submitted version.

## Conflict of Interest

The authors declare that the research was conducted in the absence of any commercial or financial relationships that could be construed as a potential conflict of interest.

## References

[1] Haupt HM Moore GW Bauer TW Hutchins GM. The lung in sickle cell disease. Chest. (1982) 81:332–7. 10.1378/chest.81.3.332 7056109

[2] Biltagi MA Bediwy AS Toema O Saeed NK. Pulmonary functions in children and adolescents with sickle cell disease. Pediatr Pulmonol. (2020) 55:2055–63. 10.1002/ppul.24871 32462802

[3] Miller GJ Serjeant GR. An assessment of lung volumes and gas transfer in sickle-cell anaemia. Thorax. (1971) 26:309–15. 10.1136/thx.26.3.309 5089498 PMC1019088

[4] Setty BY Stuart MJ Dampier C Brodecki D Allen JL. Hypoxaemia in sickle cell disease: biomarker modulation and relevance to pathophysiology. Lancet. (2003) 362:1450–5. 10.1016/S0140-6736(03)14689-2 14602439

[5] Platt OS Brambilla DJ Rosse WF Milner PF Castro O Steinberg MH. Mortality in sickle cell disease–life expectancy and risk factors for early death. N Engl J Med. (1994) 330:1639–44. 10.1056/NEJM199406093302303 7993409

[6] Mondal P Stefek B Sinharoy A Sankoorikal B-J Abu-Hasan M Aluquin V. The association of nocturnal hypoxia and an echocardiographic measure of pulmonary hypertension in children with sickle cell disease. Pediatr Res. (2019) 85:506–10. 10.1038/s41390-018-0125-6 30135591

[7] Chambellan A Dirou S Ricolleau B Graveleau J Masseau A. The value of diffusing capacity for nitric oxide and carbon monoxide in sickle cell disease. Eur Respir Soc. (2015) 46:PA516. 10.1183/13993003.congress-2015.PA516

[8] Ogilvie C Forster R Blakemore WS Morton J. A standardized breath holding technique for the clinical measurement of the diffusing capacity of the lung for carbon monoxide. J Clin Invest. (1957) 36:1–17. 10.1172/JCI103402 13398477 PMC1087291

[9] Sylvester KP Patey R Milligan P Dick M Rafferty G Rees D. Pulmonary function abnormalities in children with sickle cell disease. Thorax. (2004) 59:67–70. 14694252 PMC1758855

[10] Cotton D Graham B. Effect of ventilation and diffusion nonuniformity on DLCO (exhaled) in a lung model. J Appl Physiol. (1980) 48:648–56. 10.1152/jappl.1980.48.4.648 6769882

[11] Dinakara P Blumenthal W Johnston R Kauffman L Solnick P. The effect of anemia on pulmonary diffusing capacity with derivation of a correction equation. Am Rev Respir Dis. (1970) 102:965–9. 5486228 10.1164/arrd.1970.102.6.965

[12] Mondal P Yirinec A Midya V Sankoorikal BJ Smink G Khokhar A. Diagnostic value of spirometry vs impulse oscillometry: A comparative study in children with sickle cell disease. Pediatr Pulmonol. (2019) 54:1422–30. 10.1002/ppul.24382 31211524

[13] Chen L Gong J Matta E Morrone K Manwani D Rastogi D. Pulmonary disease burden in Hispanic and non-Hispanic children with sickle cell disease. Pediatr Pulmonol. (2020). 10.1164/ajrccm-conference.2019.199.1_MeetingAbstracts.A4387 32484996

[14] MacLean JE Atenafu E Kirby-Allen M MacLusky IB Stephens D Grasemann H. Longitudinal decline in lung volume in a population of children with sickle cell disease. Am J Respir Crit Care Med. (2008) 178:1055–9. 10.1164/rccm.200708-1219OC 18776149

[15] Lacedonia D Carpagnano GE Galgano G Schino P Correale M Brunetti ND. Usefulness of FVC/DLCO ratio to stratify the risk of mortality in patients with pulmonary hypertension. Eur Respir Soc. (2016) 48: PA2425. 10.1183/13993003.congress-2016.PA2425

[16] Matheson MC Raven J Johns DP Abramson MJ Walters EH. Associations between reduced diffusing capacity and airflow obstruction in community-based subjects. Respir Med. (2007) 101:1730–7. 10.1016/j.rmed.2007.02.020 17481877

[17] Klings ES Wyszynski DF Nolan VG Steinberg MH. Abnormal pulmonary function in adults with sickle cell anemia. Am J Respir Crit Care Med. (2006) 173:1264–9. 10.1164/rccm.200601-125OC 16556694 PMC2662970

[18] Wall MA Platt OS Strieder DJ. Lung function in children with sickle cell anemia. Am Rev Respir Dis. (1979) 120:210–4. 464381 10.1164/arrd.1979.120.1.210

[19] Koumbourlis AC Zar HJ Hurlet-Jensen A Goldberg MR. Prevalence and reversibility of lower airway obstruction in children with sickle cell disease. J Pediatr. (2001) 138:188–92. 10.1067/mpd.2001.111824 11174615

[20] Macintyre N Crapo R Viegi G Johnson D Van der Grinten C Brusasco V. Standardisation of the single-breath determination of carbon monoxide uptake in the lung. Eur Respir J. (2005) 26:720–35. 10.1183/09031936.05.00034905 16204605

[21] Larsen GL Morgan W Heldt GP Mauger DT Boehmer SJ Chinchilli VM. Impulse oscillometry versus spirometry in a long-term study of controller therapy for pediatric asthma. J Allergy Clin Immunol. (2009) 123:861–7.e1. 10.1016/j.jaci.2008.10.036 19070356 PMC4291457

[22] Thomas AN Pattison C Serjeant GR. Causes of death in sickle-cell disease in Jamaica. Br Med J. (1982) 285:633–5. 10.1136/bmj.285.6342.633 6819042 PMC1499426

[23] Hoth KF Zimmerman ME Meschede KA Arnedt JT Aloia MS. Obstructive sleep apnea. Sleep Breath. (2013) 17:811–7. 10.1007/s11325-012-0769-0 23065547 PMC3556350

[24] Ambrusko SJ Gunawardena S Sakara A Windsor B Lanford L Michelson P. Elevation of tricuspid regurgitant jet velocity, a marker for pulmonary hypertension in children with sickle cell disease. Pediatr Blood Cancer. (2006) 47:907–13. 10.1002/pbc.20791 16496290

[25] Willen SM Cohen R Rodeghier M Kirkham F Redline SS Rosen C. Age is a predictor of a small decrease in lung function in children with sickle cell anemia. Am J Hematol. (2018) 93:408–15. 10.1002/ajh.25003 29226507 PMC5803370

[26] Bishara AJ Hittner JB. Reducing bias and error in the correlation coefficient due to nonnormality. Educ Psychol Measure. (2015) 75:785–804. 10.1177/0013164414557639 29795841 PMC5965513

[27] Chen T Guestrin C. Xgboost: a scalable tree boosting system. In: Proceedings of the 22nd ACM SIGKDD International Conference on Knowledge Discovery Data Mining. San Francisco, CA (2016). 10.1145/2939672.2939785

[28] McLaren A Klingel M Behera S Odame I Kirby-Allen M Grasemann H. Effect of hydroxyurea therapy on pulmonary function in children with sickle cell anemia. Am J Respir Crit Care Med. (2017) 195:689–91. 10.1164/rccm.201606-1119LE 28248149

[29] Knight-Madden J Forrester T Lewis N Greenough A. Asthma in children with sickle cell disease and its association with acute chest syndrome. Thorax. (2005) 60:206–10. 10.1136/thx.2004.029165 15741436 PMC1747351

[30] Moreno JJM Pol AP Abad AS Blasco BC. Using the R-MAPE index as a resistant measure of forecast accuracy. Psicothema. (2013) 25:500–6. 10.7334/psicothema2013.23 24124784

[31] Wong T-T. Performance evaluation of classification algorithms by k-fold and leave-one-out cross validation. Pattern Recognit. (2015) 48:2839–46. 10.1016/j.patcog.2015.03.009

[32] Sheather S. A modern Approach to Regression With R. New York, NY: Springer Science & Business Media (2009). 10.1007/978-0-387-09608-7

[33] Liem RI Lanzkron S Coates TD DeCastro L Desai AA Ataga KI. American Society of Hematology 2019 guidelines for sickle cell disease: cardiopulmonary and kidney disease. Blood Adv. (2019) 3:3867–97. 10.1182/bloodadvances.2019000916 31794601 PMC6963257

[34] Semenova E Kameneva M Tishkov A Trofimov V Novikova L. Relationship the impulse oscillometry parameters and the lung damage in idiopathic pulmonary fibrosis patients. Eur Respir Soc. (2013) 42:1284.

[35] Mehrad B Burdick MD Wandersee NJ Shahir KS Zhang L Simpson PM. Circulating fibrocytes as biomarkers of impaired lung function in adults with sickle cell disease. Blood advances. (2017) 1:2217–24. 10.1182/bloodadvances.2017010777 29296869 PMC5737132

[36] Anthi A Machado RF Jison ML Taveira-DaSilva AM Rubin LJ Hunter L. Hemodynamic and functional assessment of patients with sickle cell disease and pulmonary hypertension. Am J Respir Crit Care Med. (2007) 175:1272–9. 10.1164/rccm.200610-1498OC 17379852 PMC2176091

[37] Sylvester KP Patey RA Milligan P Rafferty GF Broughton S Rees D. Impact of acute chest syndrome on lung function of children with sickle cell disease. J Pediatr. (2006) 149:17–22. 10.1016/j.jpeds.2005.12.059 16860119

[38] Chen G Zhang D Fuchs TA Manwani D Wagner DD Frenette PS. Heme-induced neutrophil extracellular traps contribute to the pathogenesis of sickle cell disease. Blood. (2014) 123:3818–27. 10.1182/blood-2013-10-529982 24620350 PMC4055928

[39] Zhang D Xu C Manwani D Frenette PS. Neutrophils, platelets, and inflammatory pathways at the nexus of sickle cell disease pathophysiology. Blood. (2016) 127:801–9. 10.1182/blood-2015-09-618538 26758915 PMC4760086

[40] Neas LM Schwartz J. The determinants of pulmonary diffusing capacity in a national sample of US adults. Am J Respir Crit Care Med. (1996) 153:656–64. 10.1164/ajrccm.153.2.8564114 8564114

