# Supplementary material for: Predictors of Diffusing Capacity in Children With Sickle Cell Disease: A Longitudinal Study
Source: Front Pediatr. 2021 May 31;9:678174. doi: 10.3389/fped.2021.678174 (PMC8200630; doi:10.3389/fped.2021.678174)
Supplement: Supplementary file 1 [file Data_Sheet_1.DOCX]

**Supplement File**

**1. Multicollinearity adjustment:** We estimated the degree of multicollinearity between different PFT indices based on simple linear regression analyses by including all indices in the model with hemoglobin-adjusted DLCO as the dependent variable. In this analysis, FEV1(%) had a high variance inflation factor (VIF) of 5.92 and was therefore removed from further analyses to minimize multicollinearity and stabilize the standard error estimates; the rest of the predictor variables were included in the final models for both XGBoost and regression analysis.

**2. XGBoost**

1. The aim here is to fit an XGBoost model in an optimal way such that the prediction accuracy remains high, but at the same time the model is not overfitted. The metric Root Mean Square Error (RMSE) was used to evaluate model performance.
2. For testing model performance, five-fold cross-validation (CV) was used to strike a compromise between our modest sample size and prediction accuracy. To calculate the test error, the whole sample was randomly divided in five equal folds. Next, the model was tested with one single fold of observations, whereas the remaining four folds were used in training the model. This process was repeated five times so that each fold in the original sample is used once as training data.
3. XGBoost operates through some key number of parameters that control the number of trees the algorithm grows, how long the trees should be, when to prune the trees, and how to optimize the choice between prediction accuracy and overfitting. Therefore, to achieve this aim, parameters are tuned or selected for the best possible performance. This process of tuning is known as hyperparameter optimization.
4. To choose the optimal hyperparameters which will yield the XGBoost model with lowest average cross-validated RMSE and therefore, lowest RMSE on the whole dataset, we undertook the following steps:
   1. First a list of key hyperparameters was created in such a way that the resulting model remains conservative. The list is given below

param = list(max_depth = sample(6:8, 1),

nthread = 1, verbose = F,

eta = sample(c(0.01,0.05,0.1),1),

min_child_weight=sample(c(1,2),1),

gamma = sample(c(25,50),1),

subsample = 0.5,

colsample_bytree = sample(seq(0.2,0.8,0.1),1),

colsample_bylevel= sample(seq(0.2,0.8,0.1),1),

colsample_bynode= sample(seq(0.2,0.8,0.1),1))

We described some of the key hyperparameters and our rationale behind choosing their values.

- - 1. “eta” is a scale parameter that controls the contribution of each tree. We chose smaller values of “eta” to prevent overfitting by forcing the boosting process to be more conservative.
    2. “subsample” controls the proportion of data instances to be collected in order to grow a tree. We have kept “subsample = 0.5” to prevent overfitting. Similarly, the large the value of “min_child_weight ”, the robust the model is.
    3. “gamma” is the regularization or penalty term used to make the model more conservative which works by regularizing across all the trees. We have chosen a very high value of “gamma” to increase the complexity cost of adding more leaves.

See the R page documentation of XGBoost for more finer details (<https://www.rdocumentation.org/packages/xgboost/versions/0.4-4/topics/xgb.train>)

- 1. The total combination of all the parameters in “param” list creates a grid of 20580 possibilities. Training and testing each of these 20580 choices is utterly costly. Therefore, to optimize the model fitting we randomly choose the values of parameters form “param” list.
  2. Next keeping those particular parameter values fixed, we trained the model and tested it with five fold cross validation. Each of the iterations, yielded an average RMSE. Now if the average cross validated RMSE of iteration (*i+1)* is less than that the one in iteration *(i),* the seed number is extracted and used to fit a XGBoost model on the whole dataset to obtain a whole data RMSE.
  3. If the whole data RMSE of iteration *(i+1)* is less than the one in iteration *(i)*, the seed number along with the parameter list is stored.
  4. Now this process is repeated for 10000 times and as a result we obtained 10000 RMSEs for the whole dataset. Finally, we choose the set of hyperparameter values for which the RMSE on the whole dataset is lowest.

1. The final XGBoost model with the lowest RMSE among the 10000 iterations, is given below:

set.seed(91954.47)

param = list(max_depth = sample(6:8, 1), nthread = 1, eta = sample(c(0.01,0.05,0.1),1),

min_child_weight=sample(c(1,2),1), gamma = sample(c(25,50),1),

subsample = 0.5, colsample_bytree = sample(seq(0.2,0.8,0.1),1),

colsample_bylevel= sample(seq(0.2,0.8,0.1),1), colsample_bynode= sample(seq(0.2,0.8,0.1),1))

xgboost_dlco = xgboost(data, label, nrounds = 500, params = param, verbose = F)

The resulting hyperparameters are

list(max_depth =7, nthread = 1, eta =0.1, min_child_weight=2 , gamma = 25, subsample = 0.5, colsample_bytree =0.4, colsample_bylevel=0.2, colsample_bynode= 0.8)

The final XGBoost model is robust to overfitting and conservative.

**3. Linear Mixed Effect Model**

The linear model works by using both fixed and random effects of the predictors. A random intercept was added to take into account the correlated structure for repeated measures for each patient. Parameter estimation was done based on Restricted Maximum Likelihood (REML) as implemented in R package “lme4 v1.1-23”.

**4. Missing data adjustment:**

All the study subjects had available DLCO estimates (dependent variable). Few of the predictor variables were missing at random. Assuming a Missing At Random (MAR) or Missing Completely At Random (MCAR) structure, Linear Mixed Effect Regression (LMER) models work around the missing values very efficiently. Instead of excluding any missing value or internally imputing them, LMER uses a maximum likelihood estimation approach to shrink the estimates towards the mean of the overall sample. Internally, it uses an Empirical Bayesian estimation approach to reduce the bias due to missingness. Especially in the context of longitudinal data where partial missingness is observed in covariates or exposures, LMER introduces block correlation matrices of varying sizes for each sample. Therefore, even when a particular sample includes some missing data, the shrinkage estimates and the covariance structures utilize the remaining non-missing data to obtain a robust estimate. On the other hand, XGBoost doesn’t employ any assumption of MCAR or MAR and provides the possibility to deal with missingness without any form of imputation and at the same time optimizing the prediction performance. XGBoost uses a so-called “Sparsity Aware Split Finding Algorithm” that minimizes the bias and reduces the training loss. In fact, the performance of XGBoost remains the same with or without imputing the data. (Please see four references below).

1. Molenberghs, Geert, Caroline Beunckens, Cristina Sotto, and Michael G. Kenward. 2008. “Every Missingness Not at Random Model Has a Missingness at Random Counterpart with Equal Fit.” Journal of the Royal Statistical Society: Series B (Statistical Methodology) 70 (2): 371–88. doi:10.1111/j.1467-9868.2007.00640.x.
2. Rhoads, Christopher H. 2012. “Problems with Tests of the Missingness Mechanism in Quantitative Policy Studies.” Statistics, Politics, and Policy 3 (1). doi:10.1515/2151-7509.1012.
3. Schafer, Joseph L., and John W. Graham. 2002. “Missing Data: Our View of the State of the Art.” Psychological Methods 7 (2): 147–77. doi:10.1037//1082-989X.7.2.147.
4. Rusdah, D.A., Murfi, H. XGBoost in handling missing values for life insurance risk prediction. SN Appl. Sci. 2, 1336 (2020). <https://doi.org/10.1007/s42452-020-3128-y>

**5. Mean absolute percentage error (MAPE):** “Mean absolute percentage error (MAPE): MAPE has been extensively used to estimate the accuracy of a prediction model. ‘Absolute percentage error’ is the difference between observed vs. predicted values, which is expressed as percentage of observed values. MAPE is the average of all ‘absolute percentage error’ calculated for each of the samples.

Following hypothetical example may help to understand. Three students X, Y, and Z scored 60, 70 and 80 (out of 100) respectively in Science exam. A prediction model forecasted that their scores would be 54, 84, and 92 respectively. So, in this case the ‘absolute percentage error’ for X, Y, Z would be 10%, 20%, and 15% respectively. From that information we can calculate MAPE as (10+20+15)/3=15.”

**6.** **Sample size estimation and study power:** We used comparative analyses of FVC (%) between the case vs. the control groups to estimate the minimum sample size. The FVC (mean±SD) of the controls was 105.68±19.22%. The mean FVC for the case groups was 90.34%. We calculated that 84 cases would be required to find a statistically significant difference in FVC between control and case groups (alpha=0.05, and power of 0.8), when the case to control ratio was 6:1.
