# Supplementary figures and images for: Predictors of Diffusing Capacity in Children With Sickle Cell Disease: A Longitudinal Study
Source: Front Pediatr. 2021 May 31;9:678174. doi: 10.3389/fped.2021.678174 (PMC8200630; doi:10.3389/fped.2021.678174)

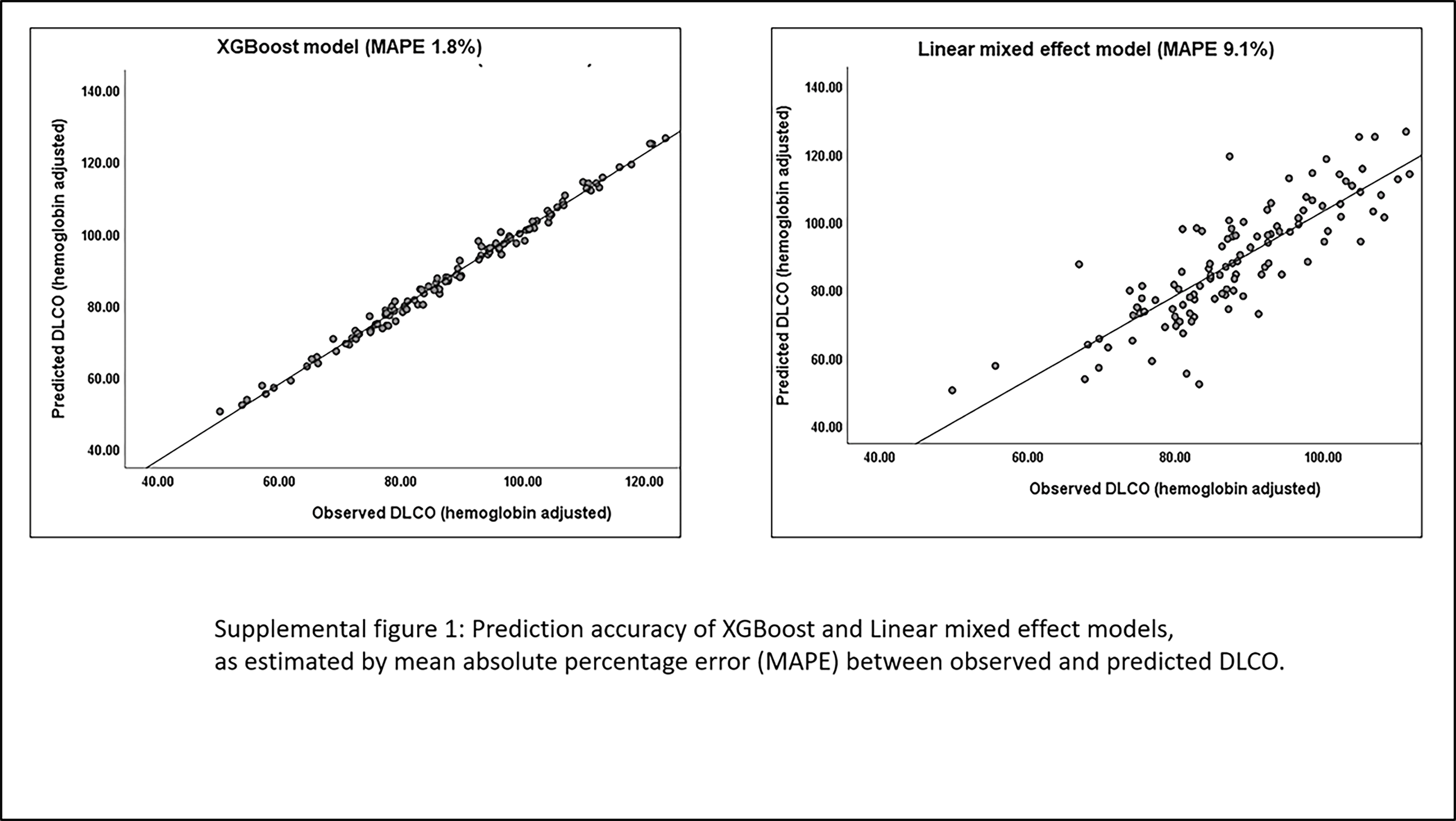

Supplement: Supplementary file 2 [file Image_1.TIF]

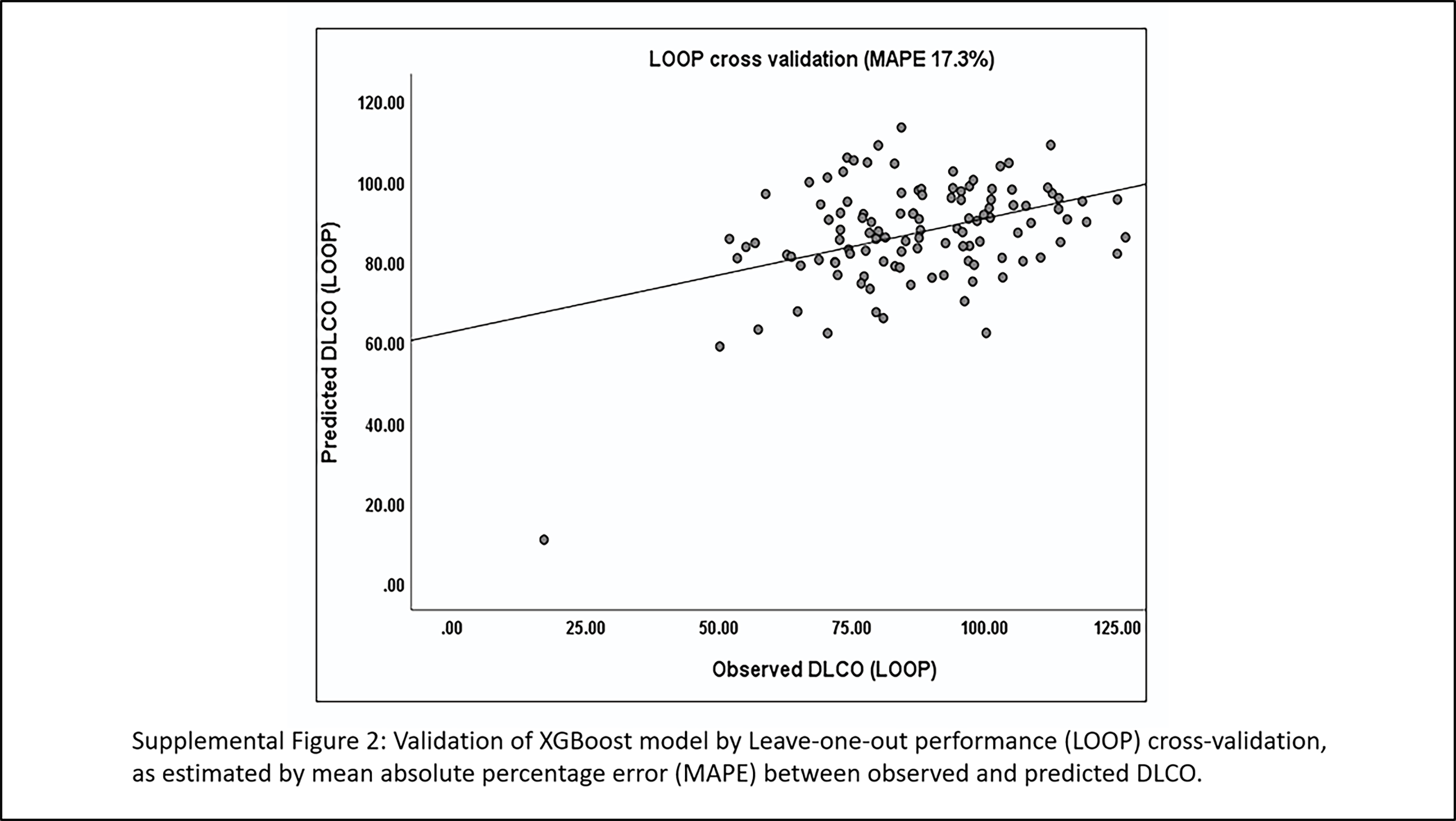

Supplement: Supplementary file 3 [file Image_2.TIF]
