# Supplementary material for: Predictors of Diffusing Capacity in Children With Sickle Cell Disease: A Longitudinal Study
Source: Front Pediatr. 2021 May 31;9:678174. doi: 10.3389/fped.2021.678174 (PMC8200630; doi:10.3389/fped.2021.678174)
Supplement: Supplementary file 4 [file Table_1.DOCX]

| **SCD biomarkers** | **N** | **Mean** | **Standard Deviation** |
| --- | --- | --- | --- |
| DLCO % predicted (Hb adjusted) | 112 | 87.92 | 17.18 |
| Total Hgb | 82 | 8.94 | 1.44 |
| HbF | 67 | 11.39 | 6.89 |
| LDH | 75 | 903.32 | 522.47 |
| Reticulocyte count | 80 | 420.38 | 725.13 |
| WBC count | 97 | 9.89 | 3.93 |
| Neutrophil (% of WBC) | 96 | 48.37 | 12.98 |
| Neutrophil count (ANC) | 96 | 4.99 | 2.72 |
| Platelet | 97 | 391.49 | 167.81 |
| BUN | 95 | 8.05 | 2.62 |
| Creatinine | 95 | 0.47 | 0.17 |
| Total Bilirubin | 94 | 3.40 | 2.72 |
| ALT | 93 | 25.80 | 13.40 |
| AST | 94 | 51.82 | 22.70 |

**e-Table 1: SCD biomarkers in the cohort (case group).**
