# Supplementary material for: Predictors of Diffusing Capacity in Children With Sickle Cell Disease: A Longitudinal Study
Source: Front Pediatr. 2021 May 31;9:678174. doi: 10.3389/fped.2021.678174 (PMC8200630; doi:10.3389/fped.2021.678174)
Supplement: Supplementary file 5 [file Table_2.DOCX]

| **Lab estimates** | **Correlations with hemoglobin- adjusted DLCO(%pred)** | | | **Correlations with total hemoglobin** | | |
| --- | --- | --- | --- | --- | --- | --- |
|  | **R** | **95% CI** | **p-value** | **R** | **95% CI** | **p-value** |
| AST | 0.258 | (0.076, 0.423) | 0.006 | -0.479 | (-0.622, -0.305) | < 0.001 |
| Total Bilirubin | 0.250 | (0.068, 0.417) | 0.008 | -0.320 | (-0.492, -0.124) | 0.002 |
| LDH | 0.198 | (0.013, 0.370) | 0.036 | -0.416 | (-0.588, -0.209) | < 0.001 |

**e-Table 2:** Association analyses among lab estimates with total hemoglobin and DLCO (%pred) adjusted for total hemoglobin and VA, respectively.

Over-adjustment Bias: AST, total bilirubin, and LDH had moderate associations with hemoglobin-adjusted DLCO(%pred). However, total hemoglobin itself had significant associations with AST, T-Bili, and LDH. The association between those lab results and hemoglobin-adjusted DLCO(%pred) was biased and primarily contributed by the correlation among lab results and with total hemoglobin. This type of error is known as over-adjustment bias. Hence, AST, T-Bili, and LDH were not included in XGBoost or regression analysis as potential predictors of adjusted DLCO.

P-values <0.05 were considered significant. R: Pearson correlation coefficient, CI: confidence interval, V_A_: alveolar ventilation.
